# Biodiversity and *γ*-Aminobutyric Acid Production by Lactic Acid Bacteria Isolated from Traditional Alpine Raw Cow's Milk Cheeses

**DOI:** 10.1155/2015/625740

**Published:** 2015-02-23

**Authors:** Elena Franciosi, Ilaria Carafa, Tiziana Nardin, Silvia Schiavon, Elisa Poznanski, Agostino Cavazza, Roberto Larcher, Kieran M. Tuohy

**Affiliations:** ^1^Research and Innovation Centre, Department of Food Quality and Nutrition, Fondazione Edmund Mach (FEM), Via E. Mach 1, 38010 San Michele all'Adige, Italy; ^2^Technology Transfer Centre, Fondazione Edmund Mach (FEM), Via E. Mach 1, 38010 San Michele all'Adige, Italy; ^3^Environmental Province Agency, Office 29.9, Laboratorio Biologico, Via Sottomonte 2, 39055 Laives, Italy

## Abstract

“*Nostrano*-cheeses” are traditional alpine cheeses made from raw cow's milk in Trentino-Alto Adige, Italy. This study identified lactic acid bacteria (LAB) developing during maturation of “*Nostrano*-cheeses” and evaluated their potential to produce *γ*-aminobutyric acid (GABA), an immunologically active compound and neurotransmitter. Cheese samples were collected on six cheese-making days, in three dairy factories located in different areas of Trentino and at different stages of cheese ripening (24 h, 15 days, and 1, 2, 3, 6, and 8 months). A total of 1,059 LAB isolates were screened using Random Amplified Polymorphic DNA-PCR (RAPD-PCR) and differentiated into 583 clusters. LAB strains from dominant clusters (*n* = 97) were genetically identified to species level by partial 16S rRNA gene sequencing. LAB species most frequently isolated were *Lactobacillus paracasei*, *Streptococcus thermophilus*, and *Leuconostoc mesenteroides*. The 97 dominant clusters were also characterized for their ability in producing GABA by high-performance liquid chromatography (HPLC). About 71% of the dominant bacteria clusters evolving during cheeses ripening were able to produce GABA. Most GABA producers were *Lactobacillus paracasei* but other GABA producing species included *Lactococcus lactis*, *Lactobacillus plantarum*, *Lactobacillus rhamnosus*, *Pediococcus pentosaceus*, and *Streptococcus thermophilus*. No *Enterococcus faecalis* or *Sc. macedonicus* isolates produced GABA. The isolate producing the highest amount of GABA (80.0±2.7 mg/kg) was a *Sc. thermophilus*.

## 1. Introduction

Traditional alpine raw milk cheeses are commonly produced in alpine regions including the province of Trentino in North-Eastern Italy. Here they are called “*Nostrano*-cheeses” and are semicooked cheese made by mixing approximately in 1 : 1 ratio the raw cow's milk from two different milking. The first milking is carried to dairy factory the evening before the cheese-making and is stored in large shallow tank for 9–11 hours where a spontaneous creaming occurs. After this overnight stage, the partially skimmed milk under the cream in the tank is manually drained from the cream fat and placed in the cheese-making vat. The whole milk from the morning milking, the second milking, is then added to the skimmed milk. No commercial lactic starters are added and the natural milk microbiota obtained from the overnight skimmed milk initiates the acidification process. The vat milk is coagulated by commercial rennet and, after the manual cutting, the curd is cooked at about 48°C. After moulding and salting, the ripening is held at about 18°C for 3 to 8 months.

The milk for “*Nostrano-*cheeses” typically comes from Holstein Friesian and/or Brown Swiss cattle breeds, which are fed differently during the year. The cows are typically fed on hay during the cold season in the valleys and from late June to middle September (summer season) are grazed on high mountain alpine pasture.

It has been reported that the use of commercial starters in raw milk cheeses may modify the characteristics of the cheese microbiota, in particular lowering the microbial biodiversity [1] and it is also well known that mainly LAB microbiota developing during ripening influences the typical organoleptic characteristics of the cheese [2]. Thus, LAB represent a fundamental process factor for the final attributes and quality of artisan dairy products such as alpine cheeses. Several studies have focused on the genotypic and technological characterization of LAB isolated from different traditionally fermented cheeses [3–6], but little work has so far been done on “*Nostrano-*cheeses.”

In addition to the technological relevance of LAB in cheese, there is currently much research and industry interest in the potential biological activity of dairy LAB, either for use as probiotics in their own wright or as bioactive agents capable of modulating the health functionality of cheese and other dairy products [7]. Raw milk cheeses have already been identified as a useful source of microbial biodiversity and new LAB strains with health promoting properties [8].

Since caseins are rich in glutamate which is released by proteolytic action, the decarboxylation of this amino acid into *γ*-aminobutyric acid (GABA) can have an important effect on the formation of eyes in cheese [9]. Besides its technological effect in cheese, GABA has several well-characterized physiological functions in mammals including neurotransmission, induction of hypotension, diuretic and tranquilizer effects, and stimulation of immune cells [10–12]. Some studies have reported also that GABA derived from the gut may be a neuroactive molecule within the gut-brain axis [13], which is a complex communication highway linking the gut environment with both the central and peripheral nervous systems. Strains of* Lb. buchneri* [14],* Lb. brevis*,* Lb. paracasei,* and* Lb. plantarum* [15] isolated from traditional cheeses have been shown to produce GABA. GABA-producing LAB have not been isolated and extensively characterised from traditional alpine cheeses produced in Trento, though the presence of GABA in these cheeses has been confirmed and its concentration at the end of ripening reported at between 120 and 1,739 mg/kg [16], which is high compared to other Italian cheese varieties (typically 0.260 to 391 mg/kg) [15]. Therefore, the objective of this study was to analyze the diversity and the successional development of LAB in traditional “*Nostrano-*cheeses” from the Trento alps during cold and summer seasons and to extensively screen and identify GABA-producing LAB isolates.

## 2. Materials and Methods

### 2.1. Cheese Factories and Milk Sampling

Cheeses were sampled in three dairy factories (called B, C, and D according to a previous paper [17]) located throughout the Trentino region and producing traditional alpine cheeses called “*Nostrano*-cheeses.” Each factory collected milk from farms within a 15 km radius.

Two cheese batches from each dairy factory, one in February and the other in July, were sampled, making a total of six batches subjected to microbiological analyses. All factories processed milk obtained from stabled cows fed with hay during the “cold season” from October to May and high mountain pasture fed cattle in the summer season from June to September. For each of the six batches, at least five cheese samples at different stages of ripening were collected (24 hours, 15 days, 1 month, 2 months, and 3 months and for five batches also 6 and 8 months) making a total of 40 cheese samples per factory.

### 2.2. Enumeration and Isolation of Microorganisms

Cheese samples (25 g) were homogenized (2 min at 260 rpm) using a stomacher (laboratory blender stomacher 400, Seward, London, UK) in 225 g peptone water (0.1% mycological peptone (Oxoid, Basingstoke, UK)) and serially diluted. Dilutions were plated and incubated as follows: onto MRS agar acidified to pH 5.5 with 5 mol/L lactic acid, anaerobically, for 2 days, at 30°C and 45°C for mesophilic and thermophilic rod-shaped LAB, respectively; onto MRS agar added with vancomycin (8 *μ*g/mL; Sigma-Aldrich, Saint Louis, MI,US) [18] and acidified to pH 5.5 with 5 mol/L lactic acid, anaerobically, for 72 h at 30°C for mesophilic heterofermentative rod-shaped LAB; onto M17 agar for 2 days, aerobically, at 30°C and anaerobically at 45°C for mesophilic and thermophilic coccoid LAB, respectively; onto KAA aerobically, for two days, at 37°C for enterococci; onto PCA added with 10 g/L skimmed milk aerobically, for 24 h, incubated at 30°C for total bacterial count (TBC). All culture media were purchased from Oxoid.

At least three colonies were picked from each countable plate; Gram-positive colonies (as determined by KOH method; [19]) and negative to the catalase test (as determined by transferring fresh colonies from agar medium to a glass slide and adding 5% H_2_O_2_) were isolated. Cell morphology was determined by microscopic observation. Each isolate was purified by subsequent culturing onto M17 or MRS and pure cultures were stored at −80°C in glycerol (20% v/v) stocks.

### 2.3. DNA Extraction and RAPD-PCR

DNA was extracted from overnight broth cultures of isolated strains. Cells were centrifuged at 10,000 ×g for 5 min and the pellets were washed twice in sterile distilled water and suspended in 1 mL of distilled water. Cell lysis was achieved using the* Instagene* Matrix (Bio-Rad, Hercules, CA, USA) following the manufacturer's instruction.

RAPD-PCR was carried out in a total volume of 25 *μ*L using primer PC1 [20]. Cluster analysis of DNA patterns was carried out using GelCompar II-BioNumerics software (package version 6.0; Applied Maths, Belgium), exploiting the unweighted pair group method arithmetic averages (UPGMA). Similarity of PCR fingerprinting profiles was calculated based on Pearson product-moment correlation coefficient. The threshold breakpoint value was fixed to 80%; isolates with similarity coefficient higher than 80% were classified into the same cluster, according to Gatti et al. [21].

### 2.4. Genotypic Identification of LAB

One isolate representative of each LAB cluster was genotypically identified by 16S rRNA gene analysis. All isolates from M17 45°C were tested by* Sc. thermophilus* species specific PCR according to Lick et al. [22]; isolates from M17 at 30°C by* Lc. lactis lactis/cremoris* species specific PCR according to Delorme et al. [23]; and all the other isolates from MRS at 30°C by* Lb. casei*,* Lb. paracasei,* and* Lb. rhamnosus *species specific PCR with the primers Y2, Casei, Para, and Rham described by Ward and Timmins [24]. If the species specific PCR gave a negative result, identification was carried out by using 16S rRNA gene sequencing. The sequence analysis of a 16S rRNA fragment gene was performed using 27f (5′-GAGAGTTTGATCCTGGCTCAG-3′) and 1495r (5′-CTACGGCTACCTTGTTACGA-3′) primers, designed by Grifoni et al. [25].

The obtained PCR products (ca. 30 ng) were purified with Exo-SAP-IT kit (USB Co., Cleveland, OH) and sequenced through the BigDye Terminator v1.1 cycle sequencing kit (Applied Biosystems, Foster City, CA) as reported by the manufacturer in an ABI PRISM 3100 sequencer (Applied Biosystems). Species were assigned after comparison of the obtained sequences by BLAST alignment (http://www.ncbi.nlm.nih.gov/BLAST).

All the amplicons were analyzed by electrophoresis on 2.5% (w/v) agarose gel (Gibco BRL, Cergy Pontoise, France) at 100 V for 90 minutes in 1X TAE buffer and were revealed by staining with ethidium bromide (0.5 *μ*g/L). All amplifications were performed with a T100 Thermal Cycler (Bio-Rad Laboratories).

### 2.5. *γ*-Aminobutyric Acid (GABA) Production and Quantification

Glutamate decarboxylase (GAD) activity of LAB isolates and the production of GABA were checked using the method of Nomura et al. [26], with some modifications; cultures were centrifuged (9,000 rpm for 15 min at 4°C), washed twice with sterile PBS, and suspended in sterile 0.85% NaCl solution in order to achieve the *A*_620 nm_ value of 2.5. Afterward; 100 *μ*L of cell suspension was mixed with 900 *μ*L of 50 mM sodium acetate buffer (pH 4.7) containing 7.0 mM L-glutamate and 0.1 mM pyridoxal phosphate. The reaction mixture was incubated for 24 h at the same temperature of isolation (30°C for mesophilic and 45°C for thermophilic isolates) and filtered through a 0.22 *μ*m pore size filter (Minisart, Sartorius Stedim Biotech, Goettingen, Germany). The sample, diluted 10 times with sodium tetraborate 0.1 M (pH adjusted to 10.5) and added to glycine, as internal standard to a final concentration of 10 mg/L, was stored at −20°C before the analysis. L-Glutamic acid, glycine, and GABA were quantified as o-phthalaldehyde (OPA) adducts modifying the method proposed by Lehtonen [27] in order to notably reduce the time of separation to only 2.7 minutes but without worsening selectivity and accuracy. This was possible in the light of the specifically designed and perfectly known matrix.

The measures were performed using an UHPLC Ultimate 3000 (Thermo Fisher Scientific, Waltham, MA, USA) equipped with a fluorescence detector (Ex = 336 nm, Em = 445 nm). Separation was carried out with sodium acetate 0.05 M (pH adjusted to 7.5; eluent A) and methanol (eluent B) using a column Chromolith Performance RP-18e (100 × 4.6 mm; Merck, Darmstadt, Germany) with Guard Cartridge Chromolith RP-18e (10 × 4.6 mm; Merck) at 40°C. The flow rate was set at 2 mL/min. The analytical gradient for eluent B was as follows: 40% for 30 sec, 25% for 90 sec, 100% for 30 sec and 60% for 15 sec. The sample (10 *μ*L), kept at 10°C by the autosampler, was automatically introduced into the loop, added with 10 *μ*L derivatising solution, mixed for 1 min, and injected. The derivatising mix was 4.5 g/L of OPA (Sigma-Aldrich) in sodium tetraborate 0.1 M, corrected to pH 10.5, 10% methanol, and 2% 2-mercaptoethanol (Sigma-Aldrich). The detection limit for GABA was estimated at 0.025 mg/L (3 times the standard deviation of the GABA contents measured repeating 10 times the analysis of a sample at unquantifiable content).

## 3. Results

### 3.1. Microbial Cell Counts

The microbial populations of* Nostrano-*cheese samples were estimated on different selective media (Table 1). The total bacterial counts were in the range of 8-9 log cfu/g from 24 h to 3 months of ripening and decreased after 6 and 8 months by 1 order of magnitude. The thermophilic cocci reached the highest counts after 24 h of ripening (mean values of 8.5 log cfu/g); mesophilic cocci reached the highest counts after 2 mo of ripening (mean values of 8.1 log cfu/g). Enterococci were never dominant and reached their highest count after 15 days of ripening (mean values of 6.2 log cfu/g). The lactobacilli group (counts onto MRS at 45 and 30°C) was higher after 1 mo of ripening. The growth dynamic of the different microbial groups was different (Table 1); thermophilic cocci counts (onto M17 45°C) were dominant in the first 24 hours; after 15 days to 3 months of ripening, mesophilic cocci (onto M17 30°C) and lactobacilli counts (onto MRS and MRS VAN 30°C) started to increase and were dominant together with thermophilic cocci; finally, at the end of ripening (6 and 8 months) mesophilic lactobacilli and thermophilic cocci maintained the dominance within the alpine cheeses microbiota.

On average, three colonies, for each colony morphology, were isolated in pure culture from each medium. For summer season at 6 and 8 months, only two types of cheese were available and sampled in dairy factories C and D. A total of 1,105 isolates were collected. From the total number of isolates, 46 were discarded from further analysis as nonlactic acid bacteria (they were found positive to catalase and negative to KOH tests). The remaining 1,059 strains were characterised for cell morphology; 677 were cocci and 382 were rods (Table 2).

### 3.2. Molecular Clustering of LAB Isolates and Species Identification

**Figure 1:**
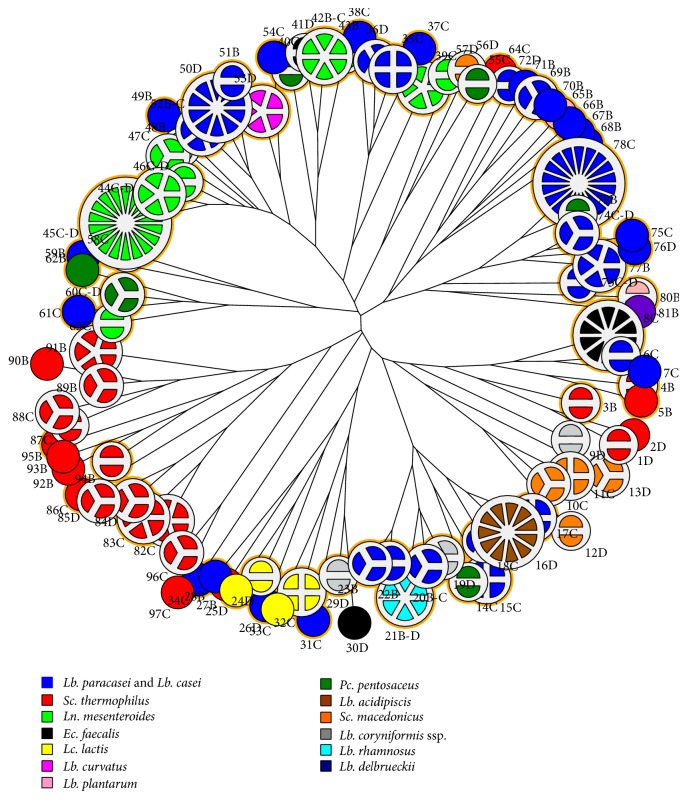
Unrooted dendrogram of the 276 dominant isolates obtained from RAPD-PCR patterns using the Pearson product moment correlation coefficient (*r*) and the unweighted pair group algorithm with arithmetic averages (UPGMA). Each circle-pie is a cluster and the number of slices represents the number of isolates for each cluster. GABA producer clusters are indicated by a yellow circle. Each color is a different species.

All putative LAB isolates were analyzed by RAPD-PCR as a first grouping into clusters. The isolates from the same kind of cheese showing a RAPD similarity coefficient of at least 80% were considered as belonging to a single cluster. The RAPD-PCR analysis grouped 1,059 LAB into 583 clusters with 80% similarity index (results not shown). From these clusters, 276 isolates were selected for further analysis because they belonged to the dominant microbial populations as enumerated by plate counts on MRS, MRS VAN at 30°C, M17 at 30, and 45°C. The RAPD-PCR analysis of these 276 dominant isolates discriminated 97 different clusters defined at a minimum similarity level of 80% (Figure 1). The 97 clusters were designated using a progressive number followed by the letters B, C, or D to indicate the dairy of origin of the clustered isolates (Figure 1; clusters 1D to 97C).

Species identification was performed by species specific PCRs or partial 16S rRNA gene sequencing.  Table 3 shows the results of bacteria identification for each ripening time. The highest diversity within a single species was found for* Lb. paracasei* and* Sc. thermophilus* with 35 and 23 different genotypes, respectively.

*Lb. paracasei* was the dominant species (72 isolated on MRS and 16 on M17 agar plates), followed by* Sc. thermophilus * (50 isolated on M17 agar) and* Ln. mesenteroides *(27 isolated on MRS and 18 on M17 agar). A different dominant species successional development was observed in cheeses at different ripening time:* Sc. thermophilus* was always dominant in the first 24 hours and one of the codominant species for up to 2 months of ripening;* Lc. lactis *species was also found codominant in cheese at 24 h ripening with* Sc. thermophilus*. Streptococci and enterococci species were recorded in abundance in the first three months but largely disappeared after six months and at the end of ripening (Table 3). We did not find difference in species distribution between cheeses sampled in February and July. The same species were recorded both in cold and in summer season (data not shown).

After the genotypic characterization, 97 strains, one representative of each dominant cluster, were processed for the detection of GABA production.

### 3.3. GABA Production

Sixty-eight isolates out of the 97 different clusters synthesized GABA (GABA amount > 0.25 mg/kg) after 24 h of incubation at 30 or 45°C in presence of glutamic acid (Table 4). They grouped 195 of the dominant isolates (71% of the tot) and in particular three (1* Lb. paracasei*, 1* Lb. rhamnosus,* and 1* Sc. thermophilus*) were able to produce GABA concentrations higher than 10 mg/L (Table 4, lines in bold).The* Sc. thermophilus*, cluster 84C showed the highest glutamate decarboxylase activity generating a mean value of 80 mg/L of GABA (Table 4, first line). No* Ec. Faecalis* or* Sc. macedonicus* isolate was able to produce amount of GABA higher than 0.25 mg/kg.

## 4. Discussion

In Italy the province of Trento has a long dairy history with various dairy biotechnological traditions arising from the geographical challenges of transport and communication between different alpine valleys and a diverse cultural heritage. A wide range of cheeses coexist, each with their own specific biotechnological processes, organoleptic characteristics, and history. A previous review has discussed the importance of preserving this type of traditional artisan cheese, usually made from raw cow's milk, because of their high microbial biodiversity and in particular high species richness of “wild” LAB with diverse metabolic activities and of great potential as dairy starters or even probiotic agents [8]. Previous work has shown that the “*Nostrano*-cheeses” contain high concentrations of GABA compared to other Italian cheeses [16] and it is known that LAB is responsible for producing GABA in cheese [15]. We, therefore, selected Trento “*Nostrano-*cheeses” for the screening and isolation of GABA-producing LAB.

The successional development of the lactic microbiota of six“*Nostrano-*cheeses” from 24 hours to 8 months of ripening was characterised. 276 isolates belonging to dominant lactic microbiota were grouped into 97 clusters, identified to the species level, and screened for their GABA production. The milk used to produce these cheeses was the subject of a previous report [17] but in summary, the microbiological characterization was in agreement with microbial counts reported for other traditional Italian cow raw milk cheeses [6, 28, 29]. M17 and MRS were not perfectly selective, in agree with previous works [6, 30] in fact some nontarget isolations were recorded; for example, 2* Lc. lactis* isolates amongst 13 were found on MRS agar plates and about 14% of all rod-shaped isolates were isolated on M17 agar plates.

As commonly found in many raw milk cheeses [28–30], the microbial composition of the “*Nostrano-*cheeses” was dominated by LAB.* Lb. paracasei* was the most abundant species (31.9% of the isolates), followed by* Sc. thermophilus* and* Ln. mesenteroides* (18.1% and 16.3%, resp.). These species were amongst the dominant microbiota at all production stages; in particular,* Sc. thermophilus* dominated after 24 h until 2 months of ripening, while* Lb. paracasei* and* Ln. mesenteroides* reached their highest levels in the cheese after 15 days and remained at high levels until 3 months of ripening with a similar trend observed in microbial counts on MRS which started to decrease after 6 months of ripening, probably the result of microbial autolysis [31, 32]. Another 11 different LAB species were found in the cheese samples but none at a relative abundance higher than 5%. All species identified were previously recorded and very common in the dairy environment [4, 6, 28–30], with the exception of* Lb. acidipiscis,* which is a species described by Tanasupawat et al. [33] and isolated from fermented fish and has also been isolated more recently from traditional Greek cheeses [34].

RAPD analysis displayed a great genetic diversity amongst the isolates. In fact about 33% of the RADP clusters were singletons (one cluster for one isolate). This genetic biodiversity may reflect a real picture of the high species richness amongst the isolates collected from the cheeses but could also be consequence of the large number of strains analysed in this study. A similar result was found in a previous work, where 206 isolates from spontaneously fermented cheeses were analysed by RAPD PCR [30].

We compared all the clusters recovered from “*Nostrano*-cheeses” with those found in the corresponding milk samples and reported in a previous report [17] and no milk RAPD pattern was found amongst the 586 cheese clusters. This may be because the fermentation is not spontaneous but driven by a starter culture from the overnight skimmed milk that, even if natural and not commercial, may inhibit milk microbiota growth and development. It is worth highlighting that some isolates from different dairies grouped within the same cluster. The 9 species occurring in different dairies were* Lb. paracasei*,* Lb. rhamnosus,* and* Ln. mesenteroides*. These few coincident clusters occurred often in different dairy environments and might represent part of an endemic geocentric cheese microbiota, not necessarily coming from milk, but adapted to the cheese-making practice, ripening, and local microclimate and environmental conditions specific to the Trento alps. On the other hand, 34 of the 97 clusters were RAPD-PCR singletons and some species like* Lb. coryniformis *ssp*. torquens, Lb. acidipiscis, Lb. curvatus,* and* Lb. delbrueckii* were peculiar only for one of the three dairy factories. These aspects suggest that each manufacturing facility may also be characterized by a unique microbial population.

Considering the recent interest in the gut-brain axis, the potential role of neurotransmitters like GABA in the periphery, and the immunological potential of systemic GABA, we screened the 97 dominant clusters for GABA producing strains [35]. A total of 68 GABA producing strains were identified. Previous studies by Siragusa et al. [15] and more recently by Diana et al. [36] found that sheep milk cheeses contained higher levels of GABA than cow's milk cheeses and consequently had higher numbers of GABA-producing LAB. The raw cow's milk cheeses subject of this current study showed higher amounts of GABA at the end of ripening [16] and a higher percent of GABA producer strains (71%) than these two previous studies where GABA producing strains were less than 14%. This difference may be due to the peculiar traditional environment of production of these Trento cheeses. However, it may also be the result of the cheese production times sampled. We screened the isolates starting at 24 h and followed the cheese LAB microbiota until the end of ripening. It is probable that microbial GABA production follows the same trend as the bacterial growth with higher number of GABA producing strains in the first 3 months followed by a rapid decrease in LAB numbers.

Amongst the 68 positive strains, 13 GABA producing strains gave more than 4 mg/kg and belonged mainly to* Lb. paracasei* species but also to* Lc. lactis*,* Lb. plantarum*,* Pc. pentosaceus, Lb. rhamnosus,* and* Sc. thermophilus*. The ability to produce GABA has been reported in various LAB, in particular* Lactobacillus* sp. isolated from fermented food [37].* Lc. lactis *and* Sc. thermophilus* were also found to produce GABA in different Italian cheeses screened by Siragusa et al. [15] and* Pc. pentosaceus* was isolated as high GABA producing strain from a Thai fermented meat [38]. From our screening, no* Ec. faecalis* or* Sc. macedonicus* strain was able to produce GABA (less than 0.25 mg/kg) and, to our knowledge, these species have never previously been identified as GABA producers.

GABA is a desired bioactive compound because of its physiological functions such as neurotransmission, induction of hypotension, diuretic and tranquilizer effects, and stimulation of immune cells [10–12]. For these beneficial effects, GABA has been introduced in the diet as an oral supplement; the Japanese government defines the foods enriched with GABA as “foods for specified health use” [3]. Fermented milk enriched in GABA produced by lactobacilli may have commercial potential as a health-oriented dairy product.

Siragusa et al. [15] observed that* Lb. paracasei*,* Lb. delbrueckii *subsp.* bulgaricus*,* Lc. lactis*,* Lb. plantarum*, and* Lb. brevis *strains isolated from different Italian cheese varieties were the best GABA-producers during the fermentation of reconstituted skimmed milk. A* Lb. casei *and* a Lc. lactis *subsp.* lactis *were used for the manufacture of a GABA-enriched fermented milk: the first strain hydrolyzed milk protein into glutamic acid and the second converted glutamic acid into GABA, respectively [39]. This current study suggests a real potential of the* Sc. thermophilus* isolate from cluster 84C to produce GABA in fermented dairy products. A daily intake of fermented milk with an amount of 10 mg of GABA for 12 weeks has been shown to decrease blood pressure by 17.4 Hg in hypertensive patients [39].* Sc. thermophilus* belonging to the cluster 84C in this current study produces 80 mg/kg of GABA. 125 mg of milk fermented with this strain could, therefore, be enough to obtain the daily intake necessary for a potential antihypertensive effect observed by Inoue et al. [39].

We are now examining the ability of this* Sc. thermophilus* strain to produce GABA in fermented milk, either alone or in association with other milk protein hydrolyzing LAB and under simulated gastrointestinal conditions.

## 5. Conclusions

This study describes the diverse lactic microbiota of traditional semihard “*Nostrano*-cheeses” from the Trento alps in Italy and how this microbiota changes during ripening. We have also characterised the potential of selected LAB isolates to produce GABA under controlled conditions, a molecule newly recognised as a putative food bioactive. We identified one* Sc. thermophilus* strain as a “high GABA producer” with considerable biotechnological potential for the development of new and attractive dairy products, an important commercial objective for increasing the potential of cheese as* multifunctional *dairy product.

## Figures and Tables

**Table 1:** Bacterial counts from cheese (*n* = 6 for each time ripening) sampled at 24 h, 15 days, 1 month, 2 months, 3 months, 6 months, and 8 months.

Ripening time	Agar media (log⁡cfu/g)						
	PCA	MRS 45	MRS 30	MRS VAN	M17 45	M17 30	KAA
24 h	8.5 ± 0.8	5.2 ± 0.6	5.3 ± 0.6	4.6 ± 1.4	8.5 ± 0.6	6.6 ± 0.6	5.6 ± 1.4
15 d	9.0 ± 0.5	5.1 ± 0.7	8.1 ± 0.8	7.7 ± 0.6	7.9 ± 1.2	7.8 ± 0.6	6.2 ± 1.0
1 month	8.8 ± 0.4	6.2 ± 1.0	8.3 ± 0.5	8.1 ± 0.5	7.8 ± 0.9	7.8 ± 0.8	5.7 ± 1.2
2 months	8.7 ± 0.3	6.3 ± 0.8	8.0 ± 0.4	8.0 ± 0.4	7.2 ± 0.7	8.1 ± 0.3	5.7 ± 1.4
3 months	8.4 ± 0.6	5.7 ± 0.7	7.8 ± 0.7	7.5 ± 0.7	7.1 ± 0.9	6.7 ± 1.6	5.7 ± 0.6
6 months^a^	7.7 ± 0.6	5.6 ± 1.5	7.0 ± 0.4	7.5 ± 0.9	6.9 ± 1.0	5.9 ± 1.4	5.7 ± 0.6
8 months^a^	6.9 ± 0.7	5.2 ± 1.0	6.5 ± 0.9	6.5 ± 0.6	6.0 ± 1.1	4.6 ± 1.5	4.6 ± 1.0

^a^ *n* = 5.

**Table 2:** Number of putative LAB strains (cocci/rods) isolated from each plate at different sampling times in traditional alpine cheeses (*n* = 6).

Ripening time	Agar media (cocci/rods)						TOT number of LAB isolates (cocci/rods)
	MRS 45	MRS 30	MRS VAN	M17 45	M17 30	KAA	
24 h	12 (7/5)	19 (15/4)	8 (8/0)	18 (18/0)	20 (18/2)	22 (21/1)	99 (88/11)
15 d	8 (8/0)	38 (10/28)	15 (1/14)	18 (17/1)	31 (24/7)	27 (22/5)	137 (82/55)
1 month	19 (10/9)	43 (15/28)	42 (19/23)	31 (28/3)	34 (28/6)	21 (20/1)	190 (120/70)
2 months	28 (16/12)	54 (17/19)	49 (20/39)	36 (35/1)	46 (33/13)	22 (19/3)	235 (140/95)
3 months	18 (13/5)	48 (21/27)	46 (24/22)	25 (24/1)	23 (22/1)	24 (17/7)	184 (121/63)
6 months^a^	8 (4/4)	30 (18/12)	25 (8/17)	16 (13/3)	22 (22/0)	12 (9/3)	113 (74/39)
8 months^a^	15 (5/10)	20 (5/15)	16 (4/12)	25 (19/6)	16 (15/1)	9 (5/4)	101 (48/48)

TOT	108 (63/45)	252 (101/151)	201 (84/117)	169 (154/15)	192 (162/30)	137 (113/24)	1059 (677/382)

^a^n = 5.

**Table 3:** Number of isolates and species found at each of the sampling time in traditional alpine cheeses (*n* = 6).

Ripening time	*Lb. paracasei *	*Sc*. *thermophilus *	*Ln*. *mesenteroides *	*Ec. faecalis *	*Lc. lactis *	*Pc*. *pentosaceus *	*Lb*. *acidipiscis *	*Sc*. *macedonicus *	*Lb. casei *	*Lb*. *coryniformis *	*Lb*. *rhamnosus *	*Lb*. *curvatus *	*Lb*. *plantarum *	*Lb*. *delbrueckii *	Total dominant isolates
24 h	—	19	—	—	6	—	—	2	1		—	—	—	—	28
15 d	20	8	3	1	1	1	—	—	4	—	2	—	3	—	43
1 month	20	10	12	3	2	2	—	—	—	—	2	5	—	—	56
2 months	14	10	13	8	—	2	—	4	3	2	2	1	—	1	60
3 months	15	3	13	1	—	5	—	5	2	3	—	—	—	—	47
6 months^a^	12	—	1	—	1	2	6	—	—	—	—	—	—	—	22
8 months^a^	7	—	3	—	3	—	5	—	—	2	—	—	—	—	20

TOT	88	50	45	13	13	12	11	11	10	7	6	6	3	1	276

^a^ *n* = 5.

**Table 4:** GABA production (Mean ± sd) of the dominant clusters in sodium acetate buffer containing 7.0 mM L-glutamate after incubation for 24 h at the same temperature of the isolation medium. Clusters are ordered from the highest to lowest GABA producer.

Cluster	Species	Isolate number	Ripening time	Isolation medium and temperature	GABA production (mg/kg)
**84C**	***Sc. thermophilus***	**3**	**24 h, 1-2 months**	**M17 45**	**80.0 ± 2.7**
**15C**	***Lb. paracasei***	**4**	**3–8 months**	**MRS 30**	**14.8 ± 5.3**
**21D-B**	***Lb. rhamnosus***	**6**	**24 h, 1-2 months**	**MRS (30, VAN)**	**11.3 ± 0.72**

24D	*Lc. lactis cremoris *	2	24 h, 6 months	M17 30	9.0 ± 0.21
62B	*Pc. pentosaceus *	1	1 month	MRS 30	6.7 ± 0.61
80B	*Lb. plantarum *	2	15 d	MRS 30	5.2 ± 0.56
59B	*Lb. paracasei *	1	15 d	MRS VAN	4.9 ± 0.20
70B	*Lb. paracasei *	1	15 d	MRS VAN	4.6 ± 0.21
22B	*Lb. paracasei *	3	15 d	MRS (30, VAN)	4.2 ± 0.41
49B	*Lb. paracasei *	1	1 month	MRS 30	4.0 ± 0.8
54C	*Lb. paracasei *	1	3 months	MRS 30	4.0 ± 0.69
73D-C	*Lb. paracasei *	3	1–6 months	M17 30, MRS 30	4.0 ± 0.29
20B-C	*Lb. paracasei *	3	15 d, 3 months	MRS 30	4.0 ± 0.18
66B	*Lb. paracasei *	1	15 d	MRS 30	4.0 ± 0.18
77D	*Lb. paracasei *	18	1-2–6 months	M17 30, MRS (30–45, VAN)	3.7 ± 0.71
68B	*Lb. casei *	3	2 months	MRS 30	3.6 ± 0.32
25D	*Lc. lactis lactis *	1	15 d	M17 30	2.8 ± 0.10
71B	*Lb. paracasei *	1	15 d	MRS VAN	2.5 ± 0.52
28B	*Lb. paracasei *	1	15 d	MRS VAN	2.5 ± 0.44
69B	*Lb. paracasei *	1	15 d	MRS VAN	2.4 ± 0.31
34C	*Lb. paracasei *	1	15 d	MRS VAN	2.3 ± 0.37
50D	*Lb. paracasei *	10	15 d, 2-3–6–8 months	M17 30, MRS (30, VAN)	2.3 ± 0.25
76D	*Lb. casei *	5	24 h, 15 d	MRS 30	2.2 ± 0.71
36C	*Lb. paracasei *	3	1-2 months	M17 30, MRS 30	2.2 ± 0.41
58C	*Lb. casei *	2	3 months	MRS VAN	2.2 ± 0.14
75C	*Lb. paracasei *	1	1 month	M17 30	2.2 ± 0.14
51B	*Lb. paracasei *	2	15 d	MRS 30	2.1 ± 0.96
72D	*Sc. thermophilus *	2	2 months	MRS 30	2.1 ± 0.11
65B	*Lb. paracasei *	1	15 d	MRS 30	1.9 ± 0.69
38C	*Lb. paracasei *	1	2 months	MRS 30	1.8 ± 0.40
79B	*Lb. paracasei *	2	1–3 months	MRS 30	1.6 ± 0.39
52B-C	*Lb. paracasei *	5	15 d, 2–6 months	MRS (30, VAN)	1.6 ± 0.32
37D	*Lb. paracasei *	1	1 month	MRS VAN	1.6 ± 0.15
4B	*Lb. paracasei *	2	2–6 months	M17 30, MRS 30	1.5 ± 0.13
78B	*Pc. pentosaceus *	2	1-2 months	MRS 30	1.46 ± 0.08
53D	*Lb. curvatus *	5	1 month	MRS (30, VAN)	1.4 ± 0.98
14C	*Pc. pentosaceus *	2	3 months	MRS VAN	1.4 ± 0.89
29D	*Lb. coryniformis ssp. torquens *	2	8 months	MRS 30	1.4 ± 0.11
35C	*Lb. paracasei *	4	1 month	MRS (30, VAN)	1.33 ± 0.066
32C	*Lc. lactis lactis *	4	24 h	MRS 30, M17 45	1.3 ± 0.37
31C	*Lb. paracasei *	1	2 months	MRS VAN	1.3 ± 0.13
17C	*Lb. paracasei *	4	2–8 months	MRS (30, VAN)	1.2 ± 0.94
74D-C	*Lb. paracasei *	1	2 months	MRS 30	1.1 ± 0.22
55C	*Pc. pentosaceus *	2	3 months	MRS 30	1.06 ± 0.044
43B	*Pc. pentosaceus *	2	6 months	MRS 30	1.00 ± 0.099
23B	*Lb. paracasei *	3	15 d, 3 months	MRS VAN	1.0 ± 0.54
67B	*Lb. paracasei *	1	1 month	MRS 30	1.0 ± 0.15
5B	*Lb. paracasei *	1	3 months	MRS 30	1.0 ± 0.12
61C	*Lb. paracasei *	2	8 months	MRS 30	0.88 ± 0.081
39C	*Ln. mesenteroides *	5	3 months	MRS (30, VAN)	0.8 ± 0.64
18C	*Lb. paracasei *	3	3 months	MRS (30, VAN)	0.8 ± 0.20
60C	*Pc. pentosaceus *	3	15 d, 2-3 months	MRS (30, VAN)	0.72 ± 0.058
19D	*Lb. coryniformis ssp. torquens *	3	2-3 months	MRS (30, VAN)	0.6 ± 0.12
95B	*Sc. thermophilus *	1	15 d	M17 45	0.6 ± 0.10
94B	*Sc. thermophilus *	2	15 d, 2 months	M17 45	0.57 ± 0.071
40C	*Ln. mesenteroides *	6	1-2 months	MRS (30, VAN)	0.57 ± 0.013
8C	*Sc. thermophilus *	1	15 d	M17 45	0.56 ± 0.045
45D-C	*Ln. mesenteroides *	18	15 d, 1-2-3–8 months	M17 30, MRS (30, VAN)	0.54 ± 0.060
3B	*Sc. thermophilus *	2	24 h	M17 45	0.52 ± 0.041
26D	*Lb. paracasei *	1	8 months	M17 45	0.50 ± 0.42
83C	*Sc. thermophilus *	5	15 d, 3 months	M17 45	0.50 ± 0.046
87C	*Sc. thermophilus *	2	24 h	M17 45	0.5 ± 0.24
44D-C	*Ln. mesenteroides *	5	2–6 months	M17 30, MRS (30, VAN)	0.48 ± 0.017
42B-C	*Ln. mesenteroides *	2	1 month	MRS 30	0.4 ± 0.13
64C	*Lb. plantarum *	1	15 d	MRS 30	0.39 ± 0.027
63C	*Ln. mesenteroides *	2	1 month	M17 30	0.37 ± 0.033
86D	*Sc. thermophilus *	1	3 months	M17 45	0.35 ± 0.055
46D-C	*Ln. mesenteroides *	2	3 months	M17 30. MRS 30	0.34 ± 0.072

1D	*Sc. thermophilus *	2	15 d	M17 45	<0.25
2D	*Sc. thermophilus *	1	15 d	M17 45	<0.25
6C	*Ec. faecalis *	10	15 d, 1-2-3 months	M17 30-45	<0.25
7C	*Sc. thermophilus *	2	24 h	M17 45	<0.25
9D	*Lb. coryniformis ssp. torquens *	2	2 months	M17 30	<0.25
10C	*Sc. macedonicus *	3	3 months	M17 45	<0.25
11C	*Sc. macedonicus *	4	2 months	M17 45	<0.25
12D	*Sc. macedonicus *	2	24 h	M17 30-45	<0.25
13D	*Sc. macedonicus *	3	8 months	M17 45	<0.25
16D	*Lb. acidipiscis *	11	6–8 months	MRS (30, VAN)	<0.25
27B	*Sc. thermophilus *	1	2 months	M17 45	<0.25
30D	*Ec. faecalis *	1	2 months	M17 30	<0.25
33C	*Lc. lactis cremoris *	1	24 h	M17 30	<0.25
41D	*Ec. faecalis *	2	2 months	M17 30	<0.25
47C	*Ln. mesenteroides *	3	15 d, 2 months	M17 30	<0.25
48B	*Lc. lactis cremoris *	2	1 month	M17 30	<0.25
56D	*Sc. macedonicus *	2	3 months	M17 45	<0.25
57D	*Ln. mesenteroides *	2	1 month	M17 30	<0.25
81B	*Lb. delbrueckii *	1	2 months	MRS 30	<0.25
82B	*Sc. thermophilus *	5	24 h, 1 month	M17 45	<0.25
85D	*Sc. thermophilus *	3	1 month	M17 45	<0.25
88C	*Sc. thermophilus *	3	2 months	M17 45	<0.25
89C	*Sc. thermophilus *	3	24 h	M17 30	<0.25
90B	*Sc. thermophilus *	1	24 h	M17 45	<0.25
91B	*Sc. thermophilus *	5	1-2 months	M17 45	<0.25
92B	*Sc. thermophilus *	1	1 month	M17 45	<0.25
93B	*Sc. thermophilus *	1	1 month	M17 45	<0.25
96B	*Sc. thermophilus *	3	24 h	M17 45	<0.25
97C	*Sc. thermophilus *	1	24 h	M17 45	<0.25
